# A Rare Case of Splenic and Pulmonary Metastases From Renal Cell Carcinoma

**DOI:** 10.7759/cureus.22914

**Published:** 2022-03-07

**Authors:** Kathie Wu, Delnaz Bakht, Priyanka Pathak, Nadia Ramdin

**Affiliations:** 1 Internal Medicine, Geisinger Medical Center, Danville, USA; 2 Hematology/Oncology, Geisinger Medical Center, Danville, USA

**Keywords:** pulmonary metastasis, nephrectomy, covid-19, splenic metastasis, renal cell carcinoma

## Abstract

Renal cell carcinoma commonly spreads to the lungs, bones, and liver, but splenic involvement has been rare. When metastasis does occur, patients are usually asymptomatic but may present with weight loss, fatigue, or abdominal pain. We present a case of a patient who had known renal cell cancer status post-total nephrectomy who, due to COVID, had delayed surveillance scans and was found to have a recurrent mass in the nephrectomy bed with splenic and pulmonary metastasis.

## Introduction

Renal cell carcinoma accounts for more than 90% of all cancers in the kidney and has been the most lethal of urologic malignancies [1]. Localized disease can be successfully managed with resection, but metastatic disease can be resistant to conventional chemotherapy, leading to the use of targeted therapies that inhibit vascular endothelial growth factor and receptor. Typical sites of spread in cases of metastatic disease include the lungs, bones, and liver. Even after primary nephrectomy, late metastases can be found up to 20 years after initial surgery and recurs in 20% to 40% of patients with previously localized disease [1-2]. Follow-up depends on the stage of disease but typically includes abdominal CT or MRI annually [3].

## Case presentation

**Figure 1:**
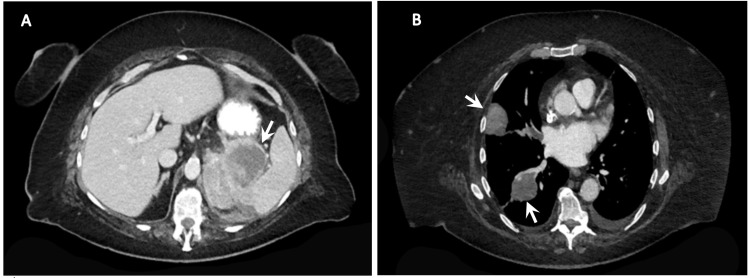
(A) Mass in the left nephrectomy bed with invasion into the splenic vasculature. (B) Presence of metastatic disease to the lungs. CT of the chest, abdomen, and pelvis with contrast demonstrating metastatic spread of renal cell carcinoma into the left nephrectomy bed and splenic vasculature and spread into the lungs.

A 72-year-old female with a past medical history of known renal cell cancer presented to office with concerns of left-sided back pain since several months. Her history was significant for a left nephrectomy a year prior for renal cell carcinoma for which she was due for a follow-up scan a year later. Due to COVID-19, her surveillance scans were delayed by several months. When they were obtained, the scans showed pulmonary nodules, which were biopsied and consistent with metastatic renal cell carcinoma for which she was started on nivolumab. A few months later, the patient presented to the office with new-onset back pain with radiation down the left leg worsening over the past several months. Given her history of metastasis, these symptoms were concerning for disease progression and she was sent for CT imaging of the chest, abdomen, and pelvis. Imaging showed a recurrent soft tissue mass in the left nephrectomy bed with invasion into the splenic vein and splenic arteries (Figure 1A) resulting in splenic infarct as well as multiple nodules and masses in bilateral lungs (Figure 1B) that increased in size, thus concerning for worsening metastatic tumor burden. Since her disease progressed despite being on nivolumab therapy and given the extent of her disease, the patient transitioned to palliative care and subsequently passed.

## Discussion

Primary and metastatic diseases to the spleen are uncommon largely due to the anatomical and histological characteristics of the spleen. The sharp angle of the splenic artery within the celiac axis along with the pulsating contractions within the sinusoids in addition to the presence of immunological cells within the spleen have been theorized to limit metastatic spread [4-5]. An estimated 30% of cases of renal cell carcinoma already have distant metastasis by the time of diagnosis, with common sites of spread to the lungs, bone, liver, and brain [6]. In these cases of metastatic spread, adjuvant therapy has only been shown to be effective in around 10% of patients [7]. However, in cases of metastasis to isolated organs, surgical resection may be performed with favorable prognosis [7].

Upon literature review, there are, to the best of our knowledge, less than 20 cases of splenic metastasis from renal cell carcinoma [4] (Table 1). In the reported cases, majority were from a left kidney primary, suggesting that there may be a degree of direct spread of tumor cells rather than hematogenous metastasis [4]. In total, 100% of patients underwent surgical resection, with two receiving adjuvant radiation or biologic therapy. All but two of the patients were alive at the time of publication, which again supports earlier surgical intervention especially in limited disease. Though rare, cases of splenic metastasis have been thought to be clinically underestimated when patients are asymptomatic. Therefore, we must rely on diagnostic imaging for surveillance as cases of renal cell metastasis can arise several years out from initial diagnosis, as seen in the literature review (Table 1).

**Table 1:** Cases of splenic metastasis originating from renal cell carcinoma in the literature.

Author	Age/Sex	Primary (Kidney)	Time to Metastasis	Treatment	Outcome
Strum, 1984 [8]	55 M	Right	264 months	Surgery and radiation	Deceased
Ishida et al., 1997 [9]	50 M	Left	84 months	Surgery	Alive
Tatsuta et al., 2001 [10]	69 M	Left	22 months	Surgery	Alive
Kugel et al., 2003 [11]	72 M	Left	24 months	Surgery	Deceased
McGregor et al., 2003 [12]	65 M	Left	Synchronous	Surgery	Unknown
Ielpo et al., 2010 [5]	68 M	Left	168 months	Surgery	Alive
Moir et al., 2011 [13]	70 F	Left	11 months	Surgery	Alive
Nunes et al., 2012 [14]	55 F	Left	60 months	Surgery	Alive
Zhang et al., 2015 [15]	67 M	Left	24 months	Surgery and radiation	Alive
Grewal et al., 2016 [16]	53 M	Left	2 months	Surgery and sunitinib	Alive
Costantini et al., 2019 [17]	41 M	Right	51 months	Surgery	Alive
Dos Santos Romao et al., 2019 [4]	48 M	Left	132 months	Surgery	Alive

## Conclusions

There are reports of renal cell carcinoma metastases to the lung but limited reports of spread to the spleen, making this case rather unusual. Given the extent of her disease, surgical resection was not pursued and the patient ultimately decided to stop all immunotherapy with transition to comfort care. This case highlights the importance of timely surveillance scans as renal cell carcinoma may present years after initial tumor diagnosis. Prompt follow-up and early diagnosis may improve overall outcomes, while disease burden remains low.
